# Oligocat: Oligoesters as Pseudo-Homogenous Catalysts for Biodiesel Synthesis

**DOI:** 10.3390/polym14010210

**Published:** 2022-01-05

**Authors:** Vitor Vlnieska, Aline Silva Muniz, Angelo Roberto dos Santos Oliveira, Maria Aparecida Ferreira César-Oliveira, Danays Kunka

**Affiliations:** 1 Chemistry Department, Federal University of Paraná (UFPR), Rua Coronel Francisco Heráclito dos Santos 100, Jardim das Américas, Curitiba 81531-980, PR, Brazil; mafco@quimica.ufpr.br (A.S.M.); arso@ufpr.br (A.R.d.S.O.); mafco@ufpr.br (M.A.F.C.-O.); 2 Swiss Federal Laboratories for Materials Science and Technology (EMPA), Überlandstrasse 129, 8600 Dubendorf, Switzerland; 3 Institute of Microstructure Technology, Karlsruhe Institute of Technology (KIT), Hermann-von-Helmholtz-Platz 1, 76344 Eggenstein-Leopoldshafen, Germany; danays.kunka@kit.edu

**Keywords:** biodiesel, second-generation feedstock, oligoesters, transesterification, fatty sources, factorial planning, homogeneous catalysis, heterogeneous catalysis

## Abstract

Biodiesel production from first-generation feedstock has shown a strong correlation with the increase in deforestation and the necessity of larger areas for land farming. Recent estimation from the European Federation for Transport and Environment evidenced that since the 2000s decade, an area equal to the Netherlands was deforested to supply global biodiesel demand, mainly originating from first-generation feedstock. Nevertheless, biodiesel is renewable, and it can be a greener source of energy than petroleum. A promising approach to make biodiesel independent from large areas of farming is to shift as much as possible the biodiesel production chain to second and third generations of feedstock. The second generation presents three main advantages, where it does not compete with the food industry, its commercial value is negligible, or none, and its usage as feedstock for biodiesel production reduces the overall waste disposal. In this manuscript, we present an oligomeric catalyst designed to be multi-functional for second-generation feedstock transesterification reactions, mainly focusing our efforts to optimize the conversion of tallow fat and sauteing oil to FAME and FAEE, applying our innovative catalyst. Named as Oligocat, our catalyst acts as a Brønsted-Lowry acid catalyst, providing protons to the reaction medium, and at the same time, with the course of the reaction, it sequesters glycerol molecules from the medium and changes its physical phase during the transesterification reaction. With this set of properties, Oligocat presents a pseudo-homogenous behavior, reducing the purification and separation steps of the biodiesel process production. Reaction conditions were optimized applying a 4^2^ factorial planning. The output parameter evaluated was the conversion rate of triacylglycerol to mono alkyl esters, measured through gel permeation chromatography (GPC). After the optimization studies, a conversion yield of 96.7 (±1.9) wt% was achieved, which allows classifying the obtained mono alkyl esters as biodiesel by ASTM D6751 or EN 14214:2003. After applying the catalyst in three reaction cycles, Oligocat still presented a conversion rate above 96.5 wt% and as well an excellent recovery rate.

## 1. Introduction

### 1.1. Biodiesel

Humanity is still highly dependent on petroleum as an energy source [1,2]. Non-renewable energies such as oil, coal and gas represent more than 80% of the total global energy consumption [3]. Several prospections have shown that the energy demand on the planet tends to increase substantially. For example, in the decade of 2030, demand for energy is projected to increase by more than 50% in comparison with the 2000s [4]. In order to reduce mainly petroleum as raw material for energy production, the usage of renewable sources might play a decisive role, which nowadays presents a humble share of 16% of the total energy consumption on our planet [5]. One of the derivate products from petroleum, the diesel fraction, can be replaced by an attractive and potentially cost-effective renewable source: biodiesel.

Biodiesel derives from environmentally friendly and renewable materials, presenting several advantages when compared with diesel fuel, for example, producing less toxic gas emissions [1], less emission of carbon monoxide (CO) and Sulphur, and its combustion has been studied, where approximately 50% or less carbon monoxide and circa 70% fewer particles, for example, sulfates, soot, nitrates, and incomplete burned hydrocarbons, are generated. Further, biodiesel is completely suitable for diesel fuel machines, not being necessary to update or adapt diesel combustion engines [6].

**Figure 1:**
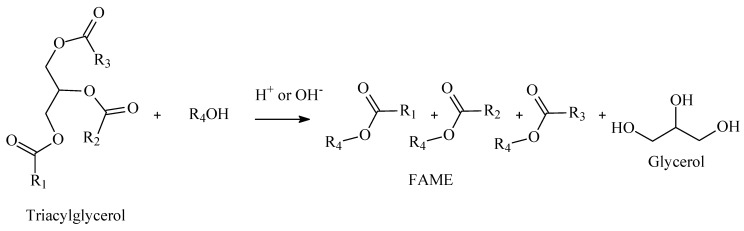
Conversion of feedstock to FAME.

The broadest definition of biodiesel is given by the American Society for Testing and Materials (ASTM), which establishes biodiesel as mono alkyl esters of long-chain fatty acids derived from renewable lipid feedstocks, such as vegetable oil or animal fat [7]. Within ASTM definition, the acronym FAME is the convention as fatty acid methyl esters, which represents the conversion product of any kind of feedstock source using methanol as an alkylation reagent. Precisely, ASTM D6751 standardizes the physical–chemical parameters that one has to comply with in order to label FAME as biodiesel. Chemically, biodiesel is simply a mono alkyl ester of a long chain structure that fulfills the quality control demanded by ASTM D6751. Figure 1 presents the general reaction for biodiesel production.

In Figure 1, labels R1, R2 and R3 represent the carbon chains from triacylglycerols, R4 represents the carbon chains of the alcohol applied for the transesterification reaction. Depending on the global region, availability and costs, other alcohols can be applied for biodiesel production, mainly ethanol, propanol and butanol, which will generate, respectively, products labeled as FAEE, FAPE and FABE. Table 1 presents the summarized acronyms for mono alkyl esters of a long chain.

For instance, using the examples of Table 1, in this work, we refer to the mono alkyl esters of the long chain as FA(X)E, which gives the freedom of choice for the alcohol as a transesterification reagent.

The kind of feedstock utilized for biodiesel production is fundamental to the chemical process, quality of the biodiesel, final costs and quantity to be produced. To date, a broad range of feedstocks have been studied in the literature, for example, almost any kind of vegetable oil, microalgae, waste sauteing oil and animal fats. Nevertheless, biodiesel still presents a higher cost when compared with diesel fuel. One of the main reasons is the utilization of edible oil sources as feedstock for its production, which is dependent on the competition with prices from the food industry, eventually resulting in feedstock trading not favorable for biodiesel production. Nevertheless, this feedstock presents the best physical–chemical properties to be applied as raw material for biodiesel production. In order to avoid the high costs of feedstock, an approach with considerable potential is the utilization of non-edible feedstock sources, for example, waste oils, animal fats and greases [1]. In literature, feedstock for biodiesel production is classified into four generations, as presented in Table 2 [4,8,9,10].

In Table 2, the first generation of feedstock is, to date, still the most applied to biodiesel production. The second generation of feedstock is fundamentally any triacylglycerol source with low commercial value, which presents, as well, the advantage of not competing with the food industry [3]. The third generation of feedstock is based on microalgae biomass technology, including the entire process chain, from cultivation and harvesting to triacylglycerol extraction, purification, and, finally, the conversion of TAG’s to FA(X)E. Usual microalgae species for biodiesel production include *Nannochloropsis* sp., *Chlorella* sp., *Scenedesmus* sp., *Pavlova lutheri*, *Iso-chrysis* sp. [10]. The fourth generation of feedstock is in an initial-phase of research, being oriented to carbon storage and capture, taking advantage of bio-engineered plants or algae [8,9]. Singh et al. 2021, describes the fourth generation biodiesel feedstock as a combination of three technologies; (i) through purpose-designed photosynthetic microorganism, (ii) through the production combination of photovoltaics and microbial fuel, and/or (iii) through synthetic cell production [4].

From the diverse sources of feedstock, within its second generation, waste sauteing oil can be considered as one of the most promising ones. Sauteing oils usually have almost no aggregated value in the market, and its usage as feedstock for biodiesel production contributes to reducing pollution and disposal transportation issues [4]. Nevertheless, one has to consider the high content of free fatty acids (FFA’s) and water, which imposes limitations on the catalytic systems and types of catalysts.

UFOP (Union zur Förderung von Oel—Und Proteinpflanzen E.V.) presented an overview of the different feedstocks applied for biodiesel production, where still circa 75% of the global production is carried out using first-generation feedstock, with 34% from palm, 26% from soybean and 16% from rapeseed oils. Second-generation feedstock represents 18% of total global production, with 11% coming from sauteing oil and 7% from animal fats [11], data are from 2019. Importantly, also to be considered is the higher demand of biodiesel nowadays. If biodiesel is continuously being produced from first-generation feedstock, larger areas of land farming will be necessary to supply this market. For example, UFOP has registered the German internal production of biodiesel since the 1990s decade, where production in 1995 was 35 × 10^3^ Tons, and in 2019 it reached 2.348 × 10^3^ Tons, an expressive increase in production volume in the last 24 years [12].

First-generation feedstock has, as well, a second major drawback. If one considers the entire process chain, including cultivation, harvesting, extraction and conversion to biodiesel, CO_2_ emissions are higher than petroleum diesel. As mentioned by the European Federation for Transport and Environment in one of its latest reports, since 2011, EU drivers have burned 39 Mt of biodiesel from palm and soy, emitting circa 381 Mt CO_2_ eq (including ILUC emissions from GLOBIUM model). This CO_2_ amount is roughly three times more if the same amount of petroleum diesel is used. The last decade presented a negative impact from biodiesel, which is produced in its majority from first-generation feedstock [13]. Notes: EU—European Union; RED—Renewable Energy Directive; ILUC—Indirect Land Use Change; GLOBIUM—Global Biosphere Management Model. The calculated life cycle emission of some feedstocks and petroleum diesel are given in Table 3 [13,14]:

In Table 3, although the value of 14 g CO_2 eq_ × MJ fuel^−1^ is not considering GLOBIUM and ILUC factors, Fotenis et al. (2020) performed the complete life cycle impact assessment (LCIA) studies for the biodiesel originating from waste sauteing oil, which presents great potential for less global CO_2_ emissions [14].

In this work, we focused our efforts to optimize the conversion of second-generation feedstock sources (tallow fat and sauteing oil) to FAME and FAEE, applying an innovative polymeric catalyst, named here as Oligocat. Reaction parameters were extensively studied in order to provide a conversion yield higher than 96.5 wt% at the first reaction cycle. To better comprehend the chemical design and optimization of Oligocat, the next section explores briefly the important characteristics that will guide the system of biodiesel production.

### 1.2. Chemical Reaction Processes for Biodiesel Production, Acid and Basic Catalisys for Transesterification of Feedstock

The five most common chemical reaction processes can be applied to convert TAG’s to FA(X)E, which are thermal cracking, transesterification and/or esterification, superfluid techniques [15], enzymatic, and Hydrotreated Vegetable Oil (HVO) techniques. HVO can be considered as a hybrid technique, which can result not only in biodiesel but in several final products, and, furthermore, biodiesel originating from HVO is usually not fully compatible with diesel machines without having engine adjustments [16]. A chemical cracking process requires high temperatures and expensive equipment. Enzymatic catalysis is difficult to scale up due to pH and temperature sensibility of the enzymes (mainly lipases), and the low solubility in alcohol usually inhibits the enzymatic activity [17]. To date, transesterification is the most applied reaction process on the biodiesel industrial scale, being able to convert TAG’s to FA(X)E within one cycle of reaction with conversion yields to fulfill either ASTM D6751 or EN 14214:2003. Nevertheless, transesterification reaction presents low kinetics velocity and is energetically unfavorable. To enhance reaction velocity, acids or bases are used as a catalyst for optimizing the chemistry process. In reality, the catalyst has to be considered as a must in order to obtain reasonable and efficient conversion yields from TAG’s to FA(X)E [7,18]. Catalyst selection is of fundamental relevance for feedstock transesterification. Basic medium reactions using homogeneous alkali catalysts, mainly sodium and potassium hydroxides, present a high-yield conversion of TAG’s to FA(X)E, with the advantages of being carried out at mild temperatures and having low costs. Nevertheless, it is most suitable for first-generation feedstock, which presents low FFAs and water contents [19,20]. As biodiesel production is still carried out in its majority by first-generation feedstock, basic catalysis is the most applied one [21].

In transesterification of TAG’s to FA(X)E, there is a biphasic system (liquid/liquid) between alcohol and the feedstock. If the catalyst is soluble in one of these phases, the system is considered homogeneous. Nevertheless, if a catalyst is insoluble in both phases, the system is considered heterogeneous. The choice of the homogeneous or heterogeneous catalytic system, being alkali or acid, plays a major role and generates operational advantages and disadvantages depending on the feedstock generation. Parameters such as reaction time, the molar ratio between alcohol and feedstock, temperature, production cost, and type of catalyst are examples that are dependent on the feedstock physical–chemical properties [22,23,24].

Regarding homogeneous catalysts, there are two major disadvantages to be considered: 1—to remove the catalyst is necessary to add a purification step in the chemical process; 2—feedstock must present a low content of FFAs and water, as previously mentioned; 3—it is difficult to recover the catalyst [25].

**Figure 2:**
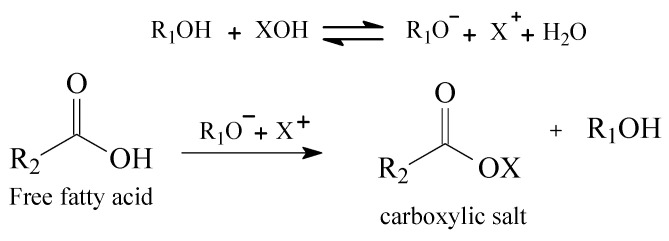
Saponification reaction of FFAs in basic medium. R_1_ represents the alcohol chain and, X stands for the alkali, and R_2_ is the FFAs chain.

In alkali mediums, FFAs are converted to carboxylic salts, which is known as saponification reaction. It reduces the FA(X)E conversion yield, mainly due to emulsification of the biphasic system, making it difficult to obtain a pure and high content of FA(X)E. Figure 2 [26,27,28] depicts the saponification reaction.

**Figure 3:**
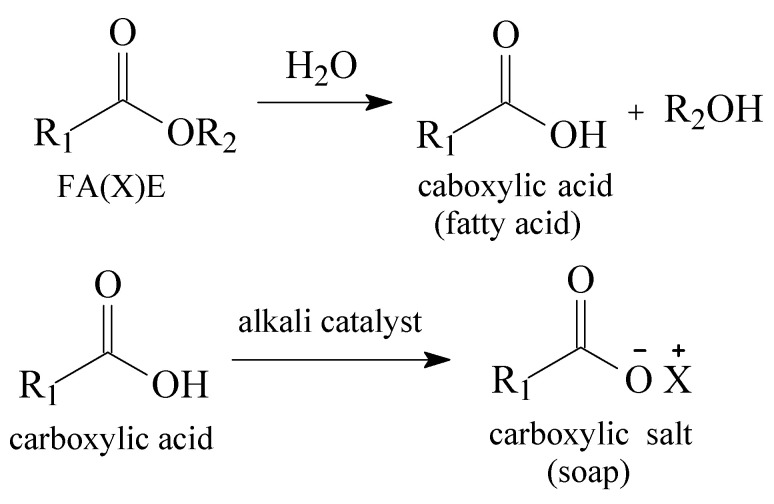
Hydrolysis reaction of FA(X)E and saponification of carboxylic acid in alkali medium.

Figure 2 represents the usual scenario of second-generation feedstock, a triacylglycerol source rich with FFAs in its composition, which prevents the application of basic catalytic systems [20]. As a quantified example, alkali medium catalytic systems shall be conducted with FFAs at a maximum of 0.5 wt% [23,29]. Important to mention as well as is the water content of the feedstock, which cannot exceed 0.3 wt% if basic catalysis is applied. Excess of water will cause hydrolyzation of the FA(X)E, leading to fatty acids and consequently being converted into carboxylic salts in basic medium, as Figure 3 depicts:

Kinetics of transesterification reaction is favored in alkali medium, being approximately four-thousand times faster than acidic catalysis, which is also one of the reasons for its application at industrial scale. Consequently, the same yield conversion can be achieved in acidic catalysis with significantly longer reaction times [23,30,31]. Table 4 presents the main characteristics of alkali and acidic mediums for transesterification reaction of TAG’s to FA(X)E.

**Figure 4:**
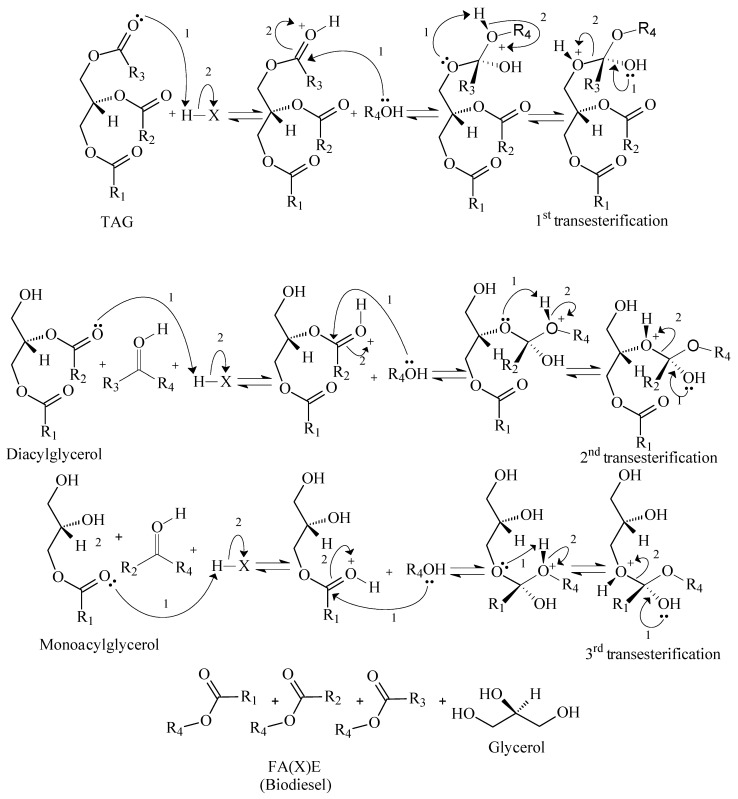
Generic acid reaction mechanism for conversion of TAG to FA(X)E.

**Figure 5:**
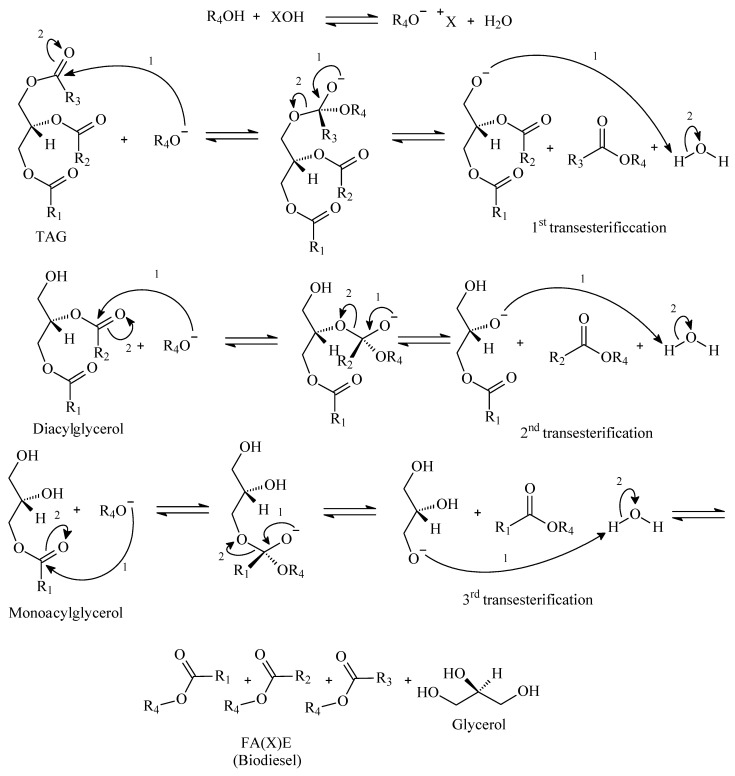
Generic basic reaction mechanism for conversion of TAG to FA(X)E.

As the main message from Figure 2 and Table 1, alkali catalysis requires strict control of the feedstock. Talley (2004) pointed out that feedstock costs for alkali catalysis can represent 60 to 75% of the final biodiesel price [32]. In acidic mediums, conversion yields above the minimum given by ASTM and EN specifications can be achieved with longer reaction times when compared with alkali medium, which is explained by the different reaction mechanisms. Figure 4 and Figure 5 present generic mechanisms for basic and acid catalytic systems.

**Figure 6:**
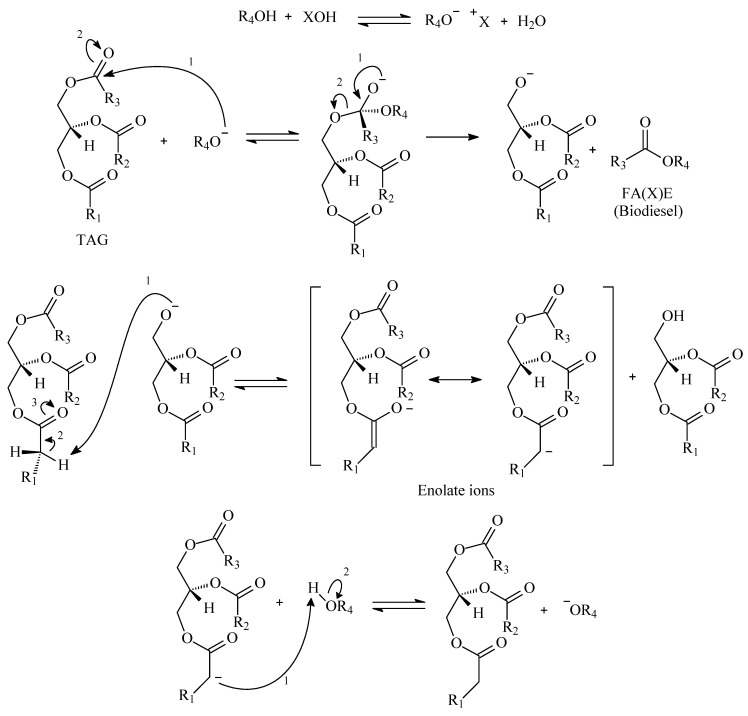
Enolate ions in the basic catalysis of TAG’s.

If Figure 4 and Figure 5 are compared, one can observe the differences between acid and basic mechanisms. In the acid mechanism, the carbonyl group has to be firstly activated by the catalyst, and afterwards, it reacts with the alcohol. In the basic mechanism, the carbonyl group reacts instantly with the alcoxy group. The alcoxy group is formed from the reaction of alcohol and catalyst, which has a faster kinetic. Some authors approached the basic mechanism for biodiesel synthesis with more detail. Dijkstra et al. (2008) suggested the formation of enolates as intermediate steps, which would increase the reaction kinetics, as Figure 6 depicts [30,33,34,35]:

Although the basic mechanism is faster, the acid mechanism is more robust. The acid mechanism is not sensitive to saponification and hydrolysation side reactions; therefore, it can be applied to any feedstock.

To summarize, Oligocat was designed following these specifications:

I—to be applied in second-generation feedstock;

II—being a Brønsted-Lowry acid catalyst;

III—to behave initially as a homogeneous catalyst;

IV—to provide conversion yield of TAG’s to FA(X)E above 96.5 wt% within one cycle of reaction.

## 2. Materials and Methods

### 2.1. Materials

Ethanol (≥99.8%), NaOH (pellets), THF (suitable for HPLC, ≥99.9%), and Ethanol (PA) were acquired from Sigma-Aldrich (Darmstadt, Germany). Glycerol trioleate, distearin, glycerol oleate (racemic mixture), and methyl oleate (GPC standards) were acquired from Aldrich (Darmstadt, Germany). Soya refined oil was acquired from Soya Ltd.a (Osasco, Brazil) and animal fat (swine) was acquired from Juliatto, Foggiatto and Cia Ltd.a (São José dos Pinhais, Brazil). All chemicals and raw materials were utilized as received. Lewatit SPC 112, a sulfonated PSDVB polymeric matrix with 12% of crosslinking and sulfonic content of 2.6 meq SO_3_H∙g^−1^ was acquired from Bayer (Leverkusen, Germany). Oligocat polymeric catalysts were previously designed, synthesized, characterized, and afterwards applied for this study.

### 2.2. Methods

#### 2.2.1. Fatty Sources Titration (AOCS Ca 5a-40)

Refined and sauteing oil: In an Erlenmeyer, 56.4 ± 0.2 g was weighed. Afterward, 50 mL of ethanol (≥99.8%) was added, and homogenization was carried out at 60 °C, in atmospheric pressure with stirring. Subsequently, the homogenized solution was titrated with a standardized solution of NaOH 0.1 mol·L^−1^.

Animal Fats: In an Erlenmeyer, 0.2 ± 0.02 g was weighed. Afterward, 30 mL of ethanol (≥99.8%) was added, and homogenization was carried out at 60 °C, in atmospheric pressure with stirring. Subsequently, the homogenized solution was titrated with a standardized solution of NaOH 0.05 mol·L^−1^.

#### 2.2.2. Determination of Alkyl Esters Conversion through Gel Permeation Chromatography (GPC)

GPC measurements were carried out in a a model 1515 (Waters, Milford, CT, USA), with two polystyrene divinylbenzene columns (PSDVB) coupled in series (TSK Gel 1000 and Styragel 100). The parameters of the GPC were: 0.8 mL·min^−1^, pressure range from 860 to 1100 bar, column oven (PE003—HPLC/GPC—Watters), and detector (Refraction index—Watters 2414) in isothermal conditions, at 40 °C.

First, a standardization curve was built, using the following compounds: Glycerol trioleate, distearin, glycerol oleate (racemic mixture), and methyl oleate. Concentration range for all compounds were from 0.25 to 8.0 mg·mL^−1^, as follows: 0.25; 0.5; 1.0; and from 1.0 to 8.0, in steps of 1.0 mg·mL^−1^, using THF (suitable for HPLC, ≥99.9%) as solvent. Afterward, all samples were weighed in the range from 2.0 to 6.0 mg·mL^−1^, using THF (suitable for HPLC, ≥99.9%) as solvent.

#### 2.2.3. Oligoesters Catalyst Recovery

After transesterification reaction, for each 100 (±0.05) mg of mixture oligoester catalyst and glycerol, an amount of 2.0 (±0.1) g of silica was used to fill the purification column (2 cm diameter). Ethanol (PA) was used as an eluent phase (15 mL). A mixture of catalyst and glycerol was separated at atmospheric pressure and room temperature. The material remaining in the eluent was dried (60 °C until constant weight) and acidulated with hydrochloric acid 0.1 mol·L^−1^ solution, washed with water until neutral pH, and once more dried until constant weight.

## 3. Results and Discussion

### 3.1. Reaction Parameters Optimization

Previously synthesized polymers poly(2,4-dihydroxy-5-sulfo-benzoic acid) (P24S), poly(2-hydroxy-5-sulfo-benzoic acid) (POS), and poly(4-hydroxy-5-sulfo-benzoic) (PPS), with circa 70 mol% of sulfonic groups functionalization (4.9 to 5.1 mmol (SO_3_H) g (polymer)^−1^), named respectively as Oligocat-P24S, Oligocat-POS and Oligocat-PPS, were evaluated as catalysts in transesterification reactions with high FFAs content. To remind, the maximum content of 0.5% in mass of FFAs is the limit to label triacylglycerol sources as a non-acidic raw matrix. For this purpose, tallow swine and used sauteing oil were investigated as potential candidates to be the raw matrix of our studies. The AOCS Ca 5a-40 method was followed to determine the acidity of those materials, and Table 5 presents the averaged triplicates of the results.

Unless under pre-treatment, both raw matrices are more suitable for the acid catalytic system. To achieve optimized reaction parameter values for the conversion of triacylglycerols into alkyl esters, a two-level factorial planning was designed, applying four variables in the reactional system. Table 3 presents variables and the values of the study, and Table 6 presents our 2^4^ factorial planning.

**Figure 7:**
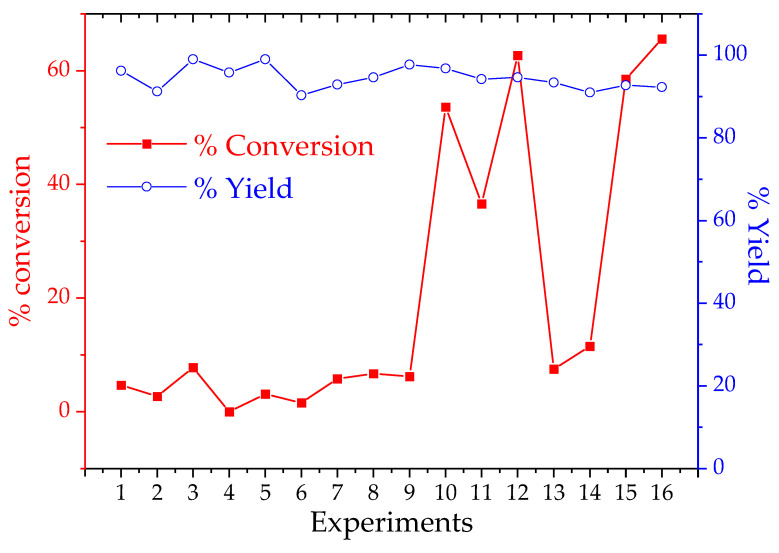
Yield and conversion obtained in a 2^4^ factorial planning for biofuel production.

In Table 7, one can see the conversion rate of triacylglycerols to alkyl esters, measured through GPC, which the results reflect the comparison of alkyl esters peak area and the sum of peak areas for tri, di, and monoacylglycerols. One can note a better output in rection 16. Nevertheless, the 65% conversion rate is not sufficient to classify the products as biofuel. In order to label an organic matrix as biofuel, it is necessary to follow the given physico–chemical characteristics from ASTM D6751, and, among other parameters, a minimum rate of 96.5% in mass composition for alkyl esters shall be achieved in the final product. In literature, the conversion rates are sought in the range from 95 to 99% [36,37]. Figure 7 presents the yield and conversion rate from the factorial planning.

In Figure 7, the yield parameter represents the conversion of triacylglycerol into any product, such as di, mono, and alkyl esters. On the other side, the conversion parameter represents only the percentage of obtained alkyl esters. Although a high yield rate was observed for the majority of experiments, presenting values higher than 90% for most of the cases, a low conversion rate was achieved, where the best result was approximately 65% of alkyl ester product. To better understand the reactional system, a synergic analysis of the variables was calculated, and a central point triplicate reaction was carried out to generate the standard deviation for the reactional system. A value of ±2.3% of conversion was obtained. Afterward, all variables were correlated in the first, second, and third levels of synergism. Table 8 represents the results of the calculation.

In Table 8, observing the first order of synergy, one can see a strong positive effect from the variable’s time, followed by the catalyst, and a slight positive effect of the temperature. The molar ratio presented no significant influence in the reactional system. The positive influence of the time, catalyst, and temperature is represented in Experiment 12, with a longer reaction time, high catalyst concentration, and high temperature, resulting in a conversion of 62.7 (±2.3)%, and Experiment 16, with the conversion of 65.6 (±2.3)%. Secondary effects of the variables were significant for temperature correlated × time, catalyst × molar ratio, and catalyst × time. Although the molar ratio does not present the first order of influence, it is interesting to note the strong influence of molar ratio × catalyst.

Observing Figure 7 once more, one can note better conversion results from reactions 9 to 16, which means experiments with longer reaction times. Observing this effect, one can reduce the factorial planning for a 2^3^ and optimize again the variable’s temperature, catalyst, and molar ratio. Table 9 presents the reduced optimization for these variables.

**Figure 8:**
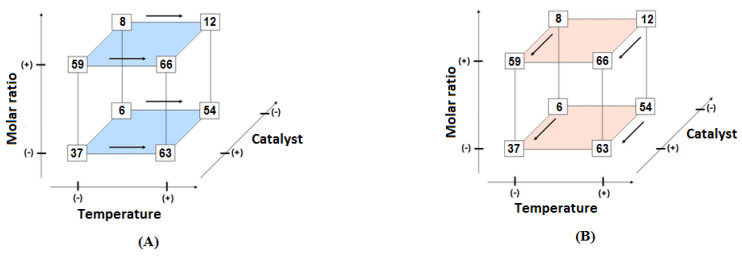
Geometric representation of an 2^3^ factorial planning, in (**A**) emphasis in temperature effects and (**B**) emphasis in catalyst concentration.

Figure 8 depicts synergic and antagonistic effects in a geometric representation. Temperature and catalyst concentration are the most significant variables for the reactional system. Nevertheless, one can see a strong secondary effect of the catalyst × molar ratio variables (synergic) and temperature × molar ratio (antagonistic effect).

**Figure 9:**
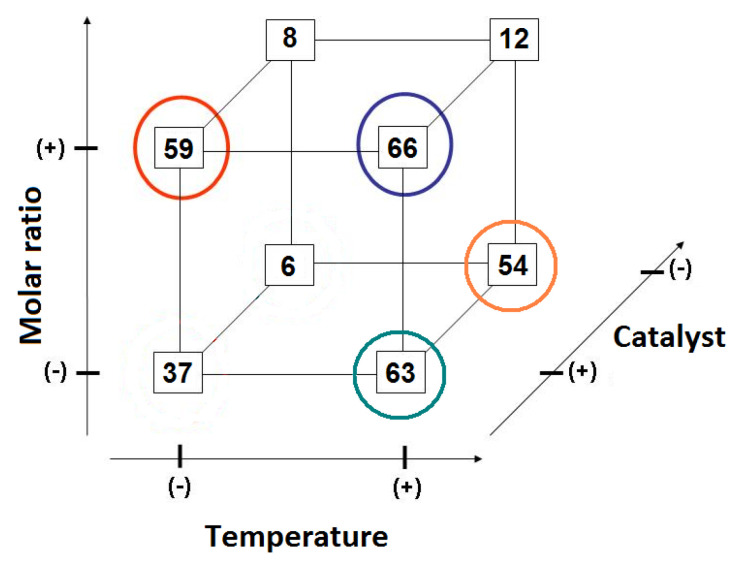
Geometric representation of 2^3^ factorial planning, with emphasis on best conversion rates.

In Figure 8A,B, the marked hyperfaces represent the molar ratio effect. In (A), one can see an improvement in the conversion rate for all geometric points from (−) to (+) level of temperature variable (indicated by the arrows). Similar behavior is observed in (B), where all geometric points presented a better conversion rate when the catalyst is used at the (+) level. Interestingly, for the molar ratio variable, going from the (−) to (+) level does not significantly improve the conversion rate. Two of the four geometrical points presented statistical equal values, and one of them presented poor conversion when compared with the (−) level. Figure 9 represents the optimal conversion rates of alkyl esters regarding the studied 2^3^ factorial planning.

In Figure 9, one can note that better conversion rates were obtained with (+) levels of temperature, catalyst, and molar ratio (dark blue circle on Figure 5), as indicated as well in the main-level-effects analysis. Nevertheless, the conversion rate of 66 (±2.3) or 63 (±2.3)% are statistically equal when ESP is considered. Still, the obtained alkyl esters cannot be classified as biodiesel, as ASTM D6751 determines [29]. To optimize conversion rates, reaction conditions were investigated at the boundaries of reaction 12, which in Figure 3 is represented by the geometrical point in a turquoise-color circle. Table 10 presents investigated reaction conditions.

Reactions 23 and 25 presented a conversion rate above the minimum standard for alkyl esters determined by ASTM D6751, which is 96.5% alkyl esters in the mass composition of the final product. ESD for this experiment was ±1.9%, resulting in statistically equal values for reactions 23 and 25. In this case, reaction 23 was chosen as the optimal reaction parameter, which uses fewer amounts of alcohol for the molar ratio variable.

### 3.2. Reutilization of Oligocat Catalysts

Oligocat catalysts were evaluated applying the optimal experimental reaction conditions presented in the previous section and, afterward, compared with the Lewatit SPC 112 as a reference catalyst. Reagents applied were tallow swine and used sauteing oil as triacylglycerol sources, using methanol and ethanol as reagent alcohols. Table 11 presents the investigated reagents and ratio conversions to alkyl esters. All conversion rates are averaged duplicates.

**Figure 10:**
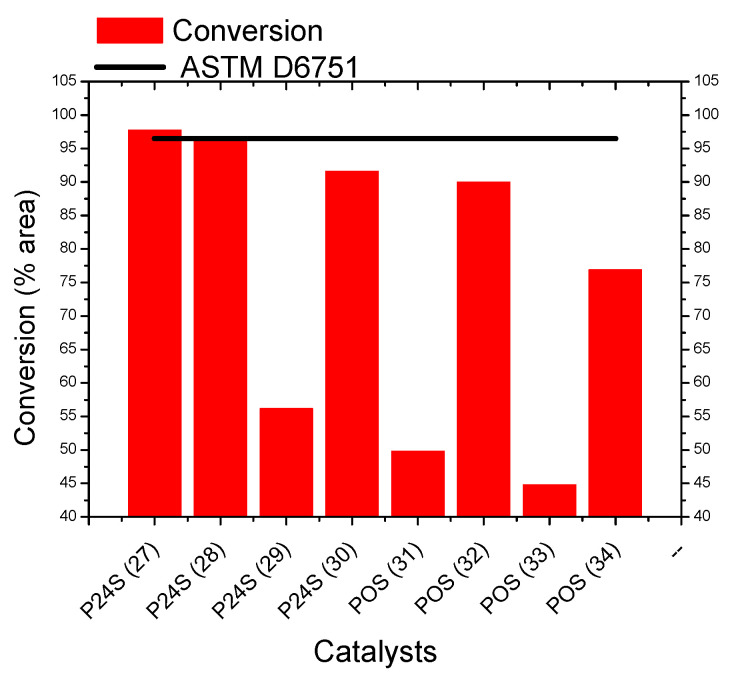
Comparison between the catalysts P24S and POS.

Oligocat catalysts were evaluated in comparison with Lewatit SPC 112 regarding the conversion of alkyl esters. Catalysts POS and P24S presented better efficiency for the optimized experimental reactional conditions, and PPS catalyst does not present significant rate conversions. Figure 10 depicts conversion rates of Oligocat POS and P24S catalysts and the minimum conversion rate imposed by ASTM D6751.

**Figure 11:**
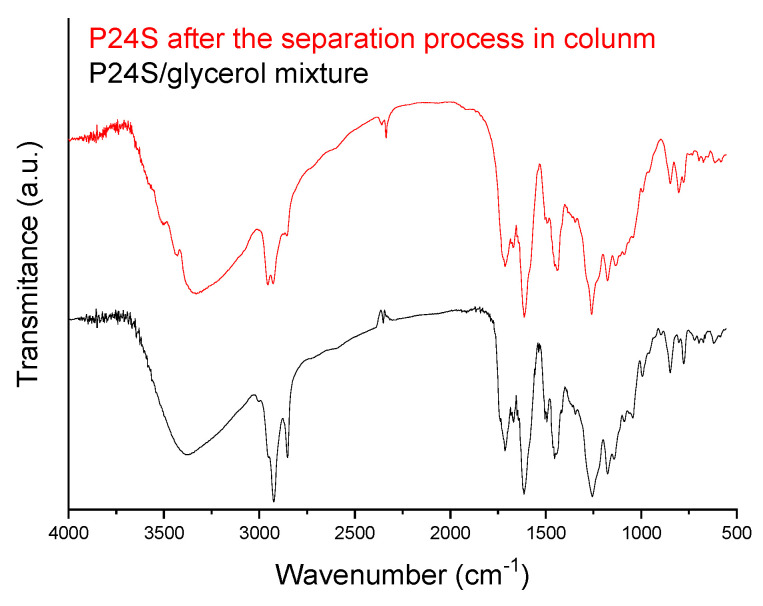
FTIR spectra before and after Oligocat P24 purification.

Oligocat P24S presented a sufficient conversion rate of alkyl esters, and the final product can be classified as biodiesel, either with ethanol or methanol, and applying tallow swine as a triacylglycerol source. Regarding sauteing oil as a triacylglycerol source, although conversion rates in the range of 90% were achieved, no catalysts were capable of complying with ASTM D6751. Interesting to note was the higher conversion rates achieved with ethanol when compared with methanol experiments. Oligocat POS and P24 were afterward evaluated in at least three cycles of reutilization. After purification of the catalyst (separation of glycerol by a conventional column filled with silica), the resultant material was evaluated by FTIR and compared with the mixture glycerol/catalyst [38]. Figure 11 present the FTIR spectra of Oligocat P24 and glycerol mixture (before purification) and FTIR spectra of Oligocat P24 purified.

In Figure 11, after the separation process, the bands in 2927 and 2850 cm^−1^ presented relative intensity reduction when compared with the bands in 1716 and 1601 cm^−1^, which indicates the separation of glycerol and catalyst (not totally) [39]. The material was acidified with HCl solution (0.1 mol.L^−1^), washed until neutral pH, dried, and reutilized in the optimal condition of the biodiesel synthesis reaction (Experiment 27—Table 11). Table 12 presents the obtained conversions to alkyl esters when the P24 was reutilized.

### 3.3. Pseudo-Homogeneous Phase Catalysts

**Figure 12:**
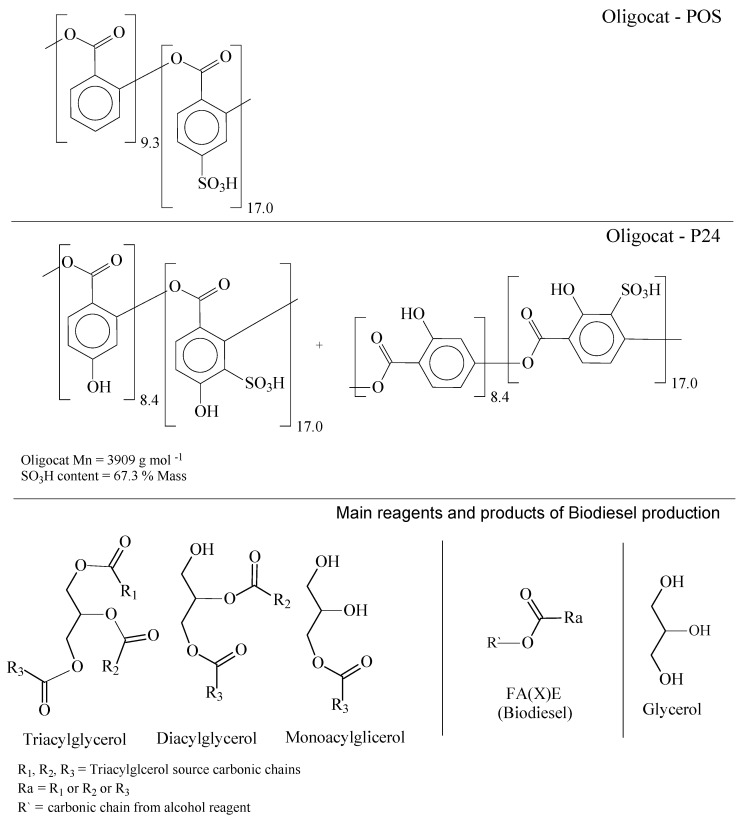
Molecular structures from Oligocat catalysts, reagents, and main products of biodiesel synthesis.

Two of the three designed catalysts presented a very interesting physical property, which was observed during the reaction course. P24 and POS catalysts sequester the glycerol molecules generated as a subproduct of triacylglycerol conversion to alkyl esters. To better comprehend such an effect, Figure 12 presents the molecular structures of P24, POS, glycerol, and the product phase.

In Figure 12, the Oligocat POS and P24 molecular structures are presented, where the polymeric backbones have an average molar mass of 3.909 × 10^3^ Da∙mol^−1^ and, as mentioned previously, are functionalized with 67.3% in mass of sulfonic groups to give the catalytic properties. Coefficients in POS and P24 represent the calculated degree of polymerization, taking into account the ratio of functionalized and non-functionalized monomeric units. The high polarity given by SO_3_H groups and the short polymeric chains can explain the good solubility of the catalysts in the alcohol phase in the initial stages of biodiesel synthesis. Nevertheless, after ending the reaction, and with the excess of alcohol being evaporated, Oligocat interacts strongly with the glycerol phase. Glycerol and Oligocat mixture present high viscosity and adheres to the reactor walls, giving the pseudo-homogenous property of Oligocat, where initially it is soluble in the alcohol phase and, afterward, together with glycerol, a viscous phase is formed and remains completely separated from the liquid states phases.

**Figure 13:**
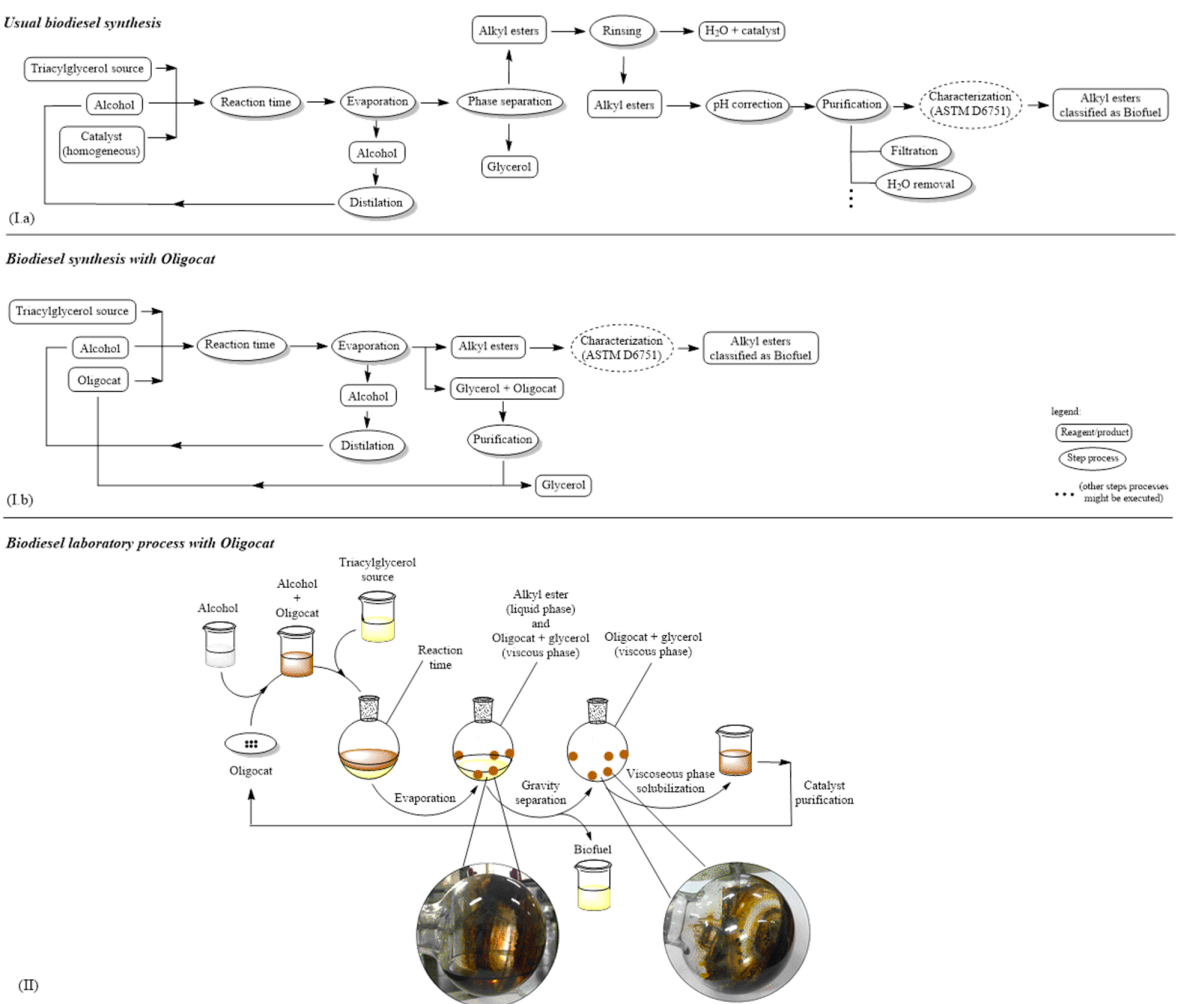
Biodiesel synthesis flow charts and laboratory synthesis steps applying Oligocat. (**Ia**) Biodiesel synthesis flow chart applying conventional homogenous alkali catalysts. (**Ib**) Biodiesel synthesis flow chart applying Oligocat. (**II**) Biodiesel reaction steps with Oligocat.

In common reaction systems with other kinds of catalysts, glycerol is usually separated by the gravity or centrifugation processes, which is time-consuming. With P24 or POS catalysts, the generated glycerol is no longer needed to be separated from the product reaction. Figure 13 presents the comparison of the usual conversion of triacylglycerol reactions and the conversion applying Oligocat catalysts.

Comparing Figure 13Ia,Ib, one can see the three main operational advantages when Oligocat is applied to biodiesel synthesis, where the first advantage is to obtain, directly, alkyl esters “ready to go” to be validated through ASTM D6751 and EN 14214:2003, where there is no need for pH correction as well as no need to remove water content, as usual homogeneous catalysts require. Still, one can see that with Oligocat, it is not necessary to wait for the phase separation between alkyl esters (biodiesel) and glycerol, after evaporation of the alcohol at the reaction system, the alkyl esters can be directly stored. In Figure 13II, one can observe the straightforward separation of the alkyl esters phase due to the pseudo-homogeneous property for this class of catalysts.

## 4. Conclusions

The necessity of further development in second-generation feedstock for biodiesel production is imminent. As literature evidenced, higher CO_2_ emissions are produced by biodiesel obtained from first-generation feedstock, which is roughly three times higher than petroleum diesel, considering GLOBIUM and ILUC factors. Larger land-farming areas are needed if one is to remain in biodiesel produced from first-generation feedstock.

Our effort in this work was to develop an acid catalyst designed for second-generation feedstock, named here as Oligocat. Through factorial planning, the biodiesel synthesis was optimized for Oligocat catalysts. POS and P24 catalysts were able to provide high conversion yields of TAG’s to FA(X)E, allowing them to be classified as biodiesel according to this parameter, which is given by ASTM D6751 and EN 14214:2003. High conversion yields above 96.5 wt% of FA(X)E were obtained applying tallow swine as feedstock, with both methanol and ethanol. For sauteing oil, using ethanol, it was possible to achieve a conversion rate in the range of 90%. A significant advantage of Oligocat is its pseudo-homogeneous property that resulted in the reduction of the operational steps for biodiesel synthesis workflow. Oligocat acts initially as a homogenous catalyst, having an optimal mass transfer of the active catalytic groups to the reaction medium. With the course of chemical reaction, Oligocat sequesters glycerol, generating a strong viscous phase, which does not interact with the biodiesel phase, resulting in fewer purification and separation steps for the biodiesel process production. With Oligocat being applied as a catalyst, there is no need to post-process the biodiesel phase. After the evaporation of the alcohol in excess from the reaction medium, Oligocat generates a biodiesel “ready to go”. The time-consuming separation phases between FA(X)E and glycerol is no longer needed. Oligocat was reutilized at least three times for biodiesel synthesis, where conversion yields above the minimum value required by ASTM D6751 and EN 14214:2003 were obtained.

## Figures and Tables

**Table 1:** Acronyms of mono alkyl esters in function of the alcohol.

Mono Alkyl Esters of Long Chains Acronym	Alcohol
FAME	Methanol
FAEE	Ethanol
FAPE	Propanol
FABE	Butanol
…	…
FA(X)E *	-

* FA(X)E: general mono alkyl ester of long chain which can be obtained from one of the alcohols used as transesterification reagent.

**Table 2:** Feedstock generation for biodiesel production.

Feedstock Generation	Examples
1	Edible oil sources: soybean, palm, rapeseed, sunflower, corn, coconut, peanut, olive
2	Non-edible oil sources: waste sautéing oil, fish oil, animal tallow oil, and grease, animal fat
3	Microalgae
4	Metabolic engineering for carbon capture and storage

**Table 3:** Life cycle emission for some biodiesel feedstocks.

Feedstock Generation	Feedstock or Petroleum Sources	Life Cycle Emission Unit: g CO_2 eq_ × MJ Fuel^−1^ (Including GLOBIUM and ILUC Factors)
-	Petroleum diesel	94
1	Biodiesel from Palm oil	282
1	Biodiesel from Soybean oil	197
2	Biodiesel from waste sauteing oil	14 *

* not including GLOBIUM and ILUC factors.

**Table 4:** Main characteristics of the alkali and acid catalysis for biodiesel production.

Alkali Catalysis	Acid Catalysis
Applicable only for feedstock with low FFAs and water contents	Applicable to any feedstock
Reaction kinetics circa four thousand times faster	Longer reaction times to achieve the same conversion yield
Less molar ratio between alcohol/feedstock	Higher molar ratio between alcohol/feedstock
FFA content: maximum 0.5 wt%	FFA content: no limit
Water content: maximum 0.3 wt%	Water content: no limit

**Table 5:** Acidity of raw materials.

Matrix	Acidity (% Mass)
Tallow swine	8.3
Used sauteing oil	0.93

**Table 6:** 2^4^ factorial planning variables and their levels.

Variable	Unit	Level (−)	Level (+)
T—Temperature	°C	60	90
C—Catalyst	% mol	5	20
R—Molar ratio	Matrix: Alcohol	1:50	1:200
Ti—Reaction time	Hours	5	48

**Table 7:** Complete 2^4^ factorial planning and measured output (conversion rate).

E	T	C	R	Ti	CR
01	60	5	1:50	5	4.7
02	90	5	1:50	5	2.7
03	60	20	1:50	5	7.8
04	90	20	1:50	5	0.0
05	60	5	1:200	5	3.0
06	90	5	1:200	5	1.6
07	60	20	1:200	5	5,8
08	90	20	1:200	5	7.0
09	60	5	1:50	48	6.2
10	90	5	1:50	48	53.6
11	60	20	1:50	48	36.6
12	90	20	1:50	48	62.7
13	60	5	1:200	48	7.5
14	90	5	1:200	48	11.5
15	60	20	1:200	48	58.5
16	90	20	1:200	48	65.6

E = Experiment; CR = Conversion rate of triacylglycerols to alkyl esters (% area); Experiments performed using Oligocat-P24S.

**Table 8:** Synergic analysis of the reaction parameters and its effects in conversion rate.

Main Effects	
Temperature	9
Catalyst	19
Molar ratio	−2
Time	34
**Second-order effects**	
Temperature × Catalyst	−2.7
Temperature × Molar ratio	−6.6
Temperature × Time	−11
Catalyst × Molar ratio	9.2
Catalyst × Time	17
Molar ratio × Time	−2
**Third-order effects**	
Temperature × Catalyst × Molar ratio	4.1
Temperature × Molar ratio × Time	9
Catalyst × Molar ratio × Time	7.2
Temperature × Catalyst × Time	−1.9
ESD *	2.3

* Experimental standard deviation with central point triplicate.

**Table 9:** Effects variables to 2^3^ planning.

First-Order Effects	
Temperature	21.1
Catalyst	36.1
Molar ratio	−4
**Second-order effects**	
Temperature × Catalyst	−4.6
Temperature × Molar ratio	−15.6
Catalyst × Molar ratio	16.4
**Third-order effects**	
Temperature × Catalyst × Molar ratio	6.1

**Table 10:** Reaction parameters in the boundaries of Experiment 12 (Table 7).

E	T	C	R	Ti	CR
17	90	10	1:5	12	28.2
18	90	10	1:25	12	56.4
19	90	15	1:25	12	58.4
20	90	10	1:75	12	29.7
21	90	15	1:75	12	38.2
12 *	90	20	1:50	48	55.5
22	90	10	1:25	36	34.3
23	90	15	1:25	36	97.6
24	90	10	1:75	36	49.1
25	90	15	1:75	36	99.0
26	90	10	1:5	36	52.3

* Experiment of Table 7, as reference.

**Table 11:** Study of other catalysts and fatty matrix in optimal condition.

E	C	FS	A	CR
27	P24S	TS	MeOH	97.8
28	P24S	TS	EtOH	96.7
29	P24S	SO	MeOH	56.2
30	P24S	SO	EtOH	91.6
31	POS	TS	MeOH	49.8
32	POS	TS	EtOH	90.0
33	POS	SO	MeOH	44.8
34	POS	SO	EtOH	76.9
39	L SPC 112	TS	MeOH	1.3
40	L SPC 112	TS	EtOH	4.4
41	L SPC 112	SO	MeOH	4.8
42	L SPC 112	SO	EtOH	5.4

E = Experiment; C = Catalyst; FS = Fatty source; A = Alcohol; CR = Conversion rate (% área); P24S = poly(2,4-dihydroxy-5-sulfo-benzoic acid); POS = poly(2-hydroxy-5-sulfo-benzoic acid); PPS = poly(4-hydroxy-5-sulfo-benzoic); TS = Tallow swine; SO = Used sauteing oil; MeOH = Methanol; EtOH = Ethanol.

**Table 12:** Reutilization of Oligocat P24S in biodiesel synthesis.

E	T	C	R	Ti	GC
28 (1° reutilization)	90	15	1:25	36	97.6
29 (2° reutilization)	90	15	1:25	36	97.3
30 (3° reutilization)	90	15	1:25	36	96.7

E = Experiment; T = Temperature (°C); C = Catalyst concentration (% mol); R = Molar ratio between “oil” and alcohol; Ti = Reaction time (horas); RC = Conversion rate (% area).

## Data Availability

Not applicable.
